# Fabrication of Lignin-Based Nano Carbon Film-Copper Foil Composite with Enhanced Thermal Conductivity

**DOI:** 10.3390/nano9121681

**Published:** 2019-11-25

**Authors:** Bin Luo, Mingchao Chi, Qingtong Zhang, Mingfu Li, Changzhou Chen, Xiluan Wang, Shuangfei Wang, Douyong Min

**Affiliations:** 1 College of Light Industry and Food Engineering, Guangxi University, Nanning 530004, China; luobinRobin123@163.com (B.L.); mingchaochi2018@163.com (M.C.); qingyutong110@163.com (Q.Z.); mingfuli@mail.gxu.cn (M.L.); chenchangzhou@gxu.edu.cn (C.C.); wangsf@gxu.edu.cn (S.W.); 2 Guangxi Key Laboratory of Clean Pulp & Papermaking and Pollution Control, Nanning 530004, China; 3 Beijing Key Laboratory of Lignocellulosic Chemistry, Beijing Forestry University, Beijing 100083, China; wangxiluan@bjfu.edu.cn

**Keywords:** lignin, copper foil, graphitization, thermal conductivity, thermal management

## Abstract

Technical lignin from pulping, an aromatic polymer with ~59% carbon content, was employed to develop novel lignin-based nano carbon thin film (LCF)-copper foil composite films for thermal management applications. A highly graphitized, nanoscale LCF (~80–100 nm in thickness) was successfully deposited on both sides of copper foil by spin coating followed by annealing treatment at 1000 °C in an argon atmosphere. The conditions of annealing significantly impacted the morphology and graphitization of LCF and the thermal conductivity of LCF-copper foil composite films. The LCF-modified copper foil exhibited an enhanced thermal conductivity of 478 W m^−1^ K^−1^ at 333 K, which was 43% higher than the copper foil counterpart. The enhanced thermal conductivity of the composite films compared with that of the copper foil was characterized by thermal infrared imaging. The thermal properties of the copper foil enhanced by LCF reveals its potential applications in the thermal management of advanced electronic products and highlights the potential high-value utility of lignin, the waste of pulping.

## 1. Introduction

Copper (Cu) is the most widely used metal in thermal management materials, owing to its relatively low cost and good theoretical thermal conductivity (K) (398 W m^−1^ K^−1)^ [1]. With the rapid development of integrated and miniaturized modern electronics, i.e., smart phones, notebooks, and wearable devices, traditional Cu materials have gradually failed to meet the requirements of rapid heat transfer. Therefore, the enhancement of the K of Cu-based composites by combining them with reinforcement materials that have a high K has captured increased attention from researchers [1,2]. A carbon-based feedstock with a high degree of graphitization possessing high theoretical K (1300–5500 W m^−1^ K^−1^) is known to be a desirable enhancer for metal-based thermal management materials [3]. Recently, a significant improvement in the K of Cu was achieved by combining it with advanced carbon enhancers including diamond, synthetic graphite (polyimide), graphene, and carbon nanotube [1,2,4,5,6]. However, the high cost and harsh production conditions of enhancers, e.g., carbon nanotubes and graphene [7,8] have limited the large-scale production of these thermal management materials. Therefore, it is more interesting, and there is market potential and research value in developing simple methodologies for fabricating carbon-Cu composite materials with high K using a wide range of renewable and inexpensive carbon precursors.

As an alternative to petrochemicals, cheap and renewable biomass resources, such as lignocellulose fiber [9], cellulose [10], lignin [11], and starch [12], have emerged as ideal carbon precursors. Among them, lignin is known to be the largest natural aromatic polymer with ~60% carbon content [13], and is usually viewed as a waste by-product and burned to provide heat for boilers. In fact, lignin has been proven to be an excellent carbon precursor owing to its high carbon content and easy graphitization [11,14]. Further, lignin has been proven to be the only natural polymer serving as a carbon precursor that can be directly converted to high-quality graphene among several amorphous carbon materials (e.g., food, cloth, paper, coal, plastic) by multiple CO_2_ laser engraving methods in an air atmosphere [15,16]. Moreover, lignin can be dissolved in some organic solvents (N, N-dimethylformamide (DMF), dimethyl sulfoxide (DMSO), acetone, acetic acid, formic acid, etc.) or water, with the possibility of generating shapeable lignin-based carbon materials using various technologies. Presently, a variety of excellent lignin-based carbon materials, including carbon fiber [17], activated carbon [18], porous carbon [19], carbon black [20], and lignin-based carbon thin film (LCF) [21], are obtained from lignin through different technologies. These lignin-based carbon materials can be used in many advanced applications such as in supercapacitors, sensors, catalysts, and thermal management materials.

Few studies have investigated lignin-based thermal management materials. However, Snowdon et al. [20] demonstrated that carbon black derived from carbonized lignin has higher degree of graphitization and higher K than commercial carbon black. Jing et al. [22] prepared lignin-based microscale carbon fibers through melt spinning followed by carbonisation at ~2500 °C; the K of the obtained lignin-based carbon fiber was found to be 24 W m^−1^ K^−1^, which is ~10 times higher than that of lignin fiber (1.83 W m^−1^ K^−1^). These studies demonstrate that lignin after carbonisation and graphitization shows a significantly improved K and great potential as a thermal management material. However, the powdery and fiber-like lignin-based carbon materials have low K values owing to poor contact between the lignin-based carbon units limiting the transmission of thermal photons.

To the best of our knowledge, there is little research focus on lignin-based carbon materials combined with copper to synthesize metal-based composite films for thermal management application. Herein, a novel, low-cost, carbon-copper composite film with enhanced K from lignin and Cu foil via a practical method is reported in current study. Dense nanoscale lignin-based carbon thin films with a high carbon content and high degree of graphitization were obtained by simple spin coating followed by annealing treatment. The effect of the annealing residence times (RET) at 1000 °C and cooling time (CT) on the appearance, structure, carbon content, graphitization degree, and thermal properties of lignin-based carbon films were studied in the present work. This work not only provides a practical way to develop low-cost and effective thermal management materials, but also demonstrates promising prospects of high-valued applications of lignin.

## 2. Materials and Methods

### 2.1. Materials

Kraft pulping black liquor of sugarcane bagasse was kindly supplied by Guitang Co. Ltd. (Guangxi, China). The solid content of black liquor is 45%, in which the content of lignin, ash, lignin carbohydrate complex, polysaccharides and other ingredients were 26.7%, 42.2%, 4.4%, 17.8%, and 8.9%, respectively. Ethanol (AR grade), acetone (AR grade), and ammonium persulfate (AR grade) were purchased from Nanning Lantian Company (Guangxi, China). The Cu foils (25 μm thickness, product # 010950) were purchased from Alfa Aesar Chemical Company (Shanghai, China).

### 2.2. Preparation of Lignin-Based Carbon Film-Copper Foil Composite

The black liquor was acidified to pH = 2 by acetic acid to obtain the crude lignin. Then, the impurities were removed by dissolving lignin in acetone followed by centrifugation at 5000 r/min for 20 min. The purified lignin was recovered by the rotary evaporation of the resultant supernatant. Then, a 0.2% (*w*/*v*) purified lignin/acetone spin coating solution was obtained by dissolving 200 mg of purified lignin in 100 mL of acetone. 200 µL of purified lignin/acetone solution was applied on the surfaces of Cu foil (ϕ 25 mm) for spin coating. The spin coating was completed at 5000 rpm for 1 min at 25 °C. After the complete removal of acetone from the lignin thin film in an oven at 70 °C for 2 h, the other surface of the Cu foil was coated with a thin film of lignin via the same procedure.

The Cu foils coated with lignin thin films (LF) were heated to 1000 °C at the heating rate of 10 °C/min under argon atmosphere with 200 mL/min argon flow rate in a tube furnace. Two RET, 20 min and 120 min, were employed for the annealing treatment of the samples at 1000 °C. Three CT from 1000 °C to room temperature of 40, 150, and 300 min were investigated. The annealed samples are accordingly named as LCF–Cu _a–b_, where a represents RET, and b represents CT. The Cu foil was also annealed at 1000 °C for 120 min RET and 300 min CT and used as a reference.

### 2.3. Characterizations

#### 2.3.1. Morphology and Structure of Lignin-Based Carbon

The morphology of the samples was characterized by scanning electron microscopy with 5 kV (SEM, Phenom F16502, Eindhoven, Holland). Atomic force microscopy (AFM, Hitachi 5100N, Tokyo, Japan) was conducted using scanning probe microscope in tapping mode. Transmission electron microscopy (HRTEM), and selected area electron diffraction (SAED) were carried out on a 300 kV field-emission transmission electron microscope (Tecnail G2F20, FEI, Hillsboro, OR, USA). X-ray photoelectron spectroscopy (XPS) was performed on a photoelectron spectrometer (XPS, Escalab 250XL+, Thermo fisher scientific, Waltham, MA, USA) using Al Kα (1486.6 eV) radiation. Raman spectra were recorded on a micro Raman spectrometer (Horiba, JYH-800, Paris, France) using a 488 nm laser as the excitation source.

#### 2.3.2. Thermal Conductivity of Lignin-Based Carbon Film-Copper Foil Composite

Thermal diffusivity (α) was measured by a laser flash technique using a NETZSCH LFA 447 Nano Flash^TM^ (NETZSCH, Selb, Germany) diffusivity apparatus. The parameters of α measurement were: voltage, 250 V; pulse width, 0.05 ms. Each measurement of α was replicated three times and the average value was reported. The relative standard deviation of each series of measurements was less than 2.6%. The specific heat (C_p_) of the samples was determined by differential scanning calorimetry (NETZSCH DSC 204, Selb, Germany) at a heating rate of 10 °C/min in N_2_ atmosphere. The infrared images were captured using the thermal emission microscopy system (Optotherm Sentris IS640, Sewickley, PA, USA).

## 3. Results and Discussion

### 3.1. Effect of Annealing Conditions on Morphology and Structure of Lignin-Based Carbon Film

Low toxic and volatile acetone was used for preparing the spin coating lignin solution [23]. Lignin, as a thermoplastic macromolecule with glass transition temperature at about 180 °C, can be melted during annealing at a high temperature, resulting in the reconstitution of the lignin film [24]. The effect of different temperatures on the graphitization of biomass-derived carbon materials was reported [25]. It is revealed that the higher the temperature applied is, the higher the graphitization degree of the biomass-based carbon materials is [26]. However, the effects of RET and CT on the degree of graphitization and carbon content have been rarely studied. Therefore, the LF was annealed under argon atmosphere at 1000 °C with different RETs and CTs. Not only was the K was enhanced through the graphitization of lignin by the copper as catalyst, but also the morphology became dense for heat transfer.

#### 3.1.1. SEM Analysis

**Figure 1:**
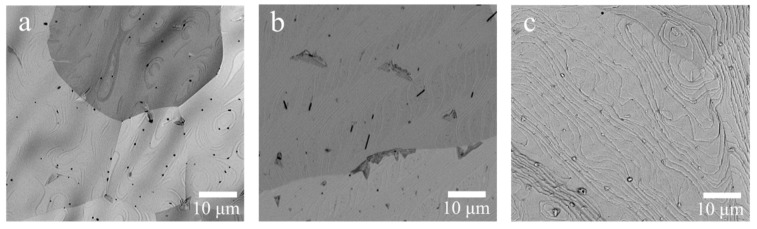
SEM images of lignin carbon thin film (LCF) on the surface of copper foil: (**a**) LCF–Cu _20–40_, (**b**) LCF–Cu _120–40_, and (**c**) LCF–Cu _120–300_.

During the spinning process, acetone was easily evaporated, leaving behind a solid lignin thin film on the surface of the Cu foil. However, the rapid evaporation of acetone led to a porous structure of the lignin film (Figure S1). Figure 1a–c shows LCF–Cu exhibits a dense surface with no pore structure as the annealing temperature was applied at 1000 °C. The RET and CT in the conditions of annealing significantly affected the degree of graphitization and the final morphology of the lignin-based carbon material [27]. When the annealing was performed with short RET and CT, the surface morphology of LCF–Cu _20–40_ was found to be relatively smooth with a small quantity of carbon particles (Figure 1a). This can be explained as follows: free carbon atoms were generated in the initial stage of the carbonisation at the high temperature, and they continuously aggregated to form a crystalline carbon film or a small quantity of carbon particles [25]. A relatively smoother surface was observed for LCF–Cu _120–40_, suggesting that more carbon atoms participated in the formation of the carbon film with the extension of RET (Figure 1b). When the annealing treatment was conducted with the longest RET and CT, a host of obvious ripples were observed on the surface of LCF–Cu _120–300_ (Figure 1c), and the shape of these ripples was found to be similar to that of graphene obtained by chemical vapour deposition (CVD) methods under the catalysis of Cu foil. A curtain of ripples may be produced from interfacial instabilities driven by capillary forces in solution [25]. This indicated the appearance of highly graphitized and dense structure of the LCF is mainly due to effect of reduced cooling rate on the aggregation of carbon atoms. The results revealed the formation of a dense LCF by the melting and reconstitution of lignin molecules followed by their graphitization through the loss of O and H atoms during the annealing treatment with the extension of RET and CT at 1000 °C. SEM showed that LCF–Cu _120–300_ had the most complete film structure. In order to further study the structure and morphology of LCF–Cu _120–300_, TEM and AFM were also employed.

#### 3.1.2. TEM and AFM Analysis

**Figure 2:**
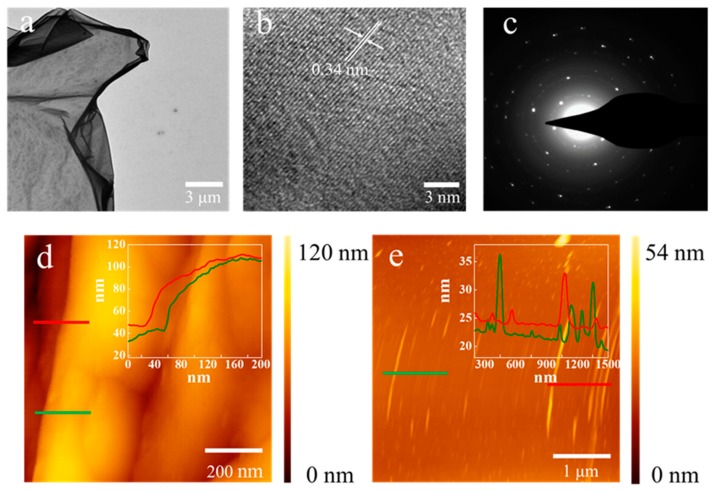
(**a**) TEM image of LCF–Cu _120–300_; (**b**) HRTEM images at different positions for LCF–Cu _120–300_, (**c**) the corresponding SAED image; (**d**) AFM image showing thickness of LCF; (**e**) AFM image showing height of the ripples.

TEM and AFM were also conducted to characterize the microstructure and thickness of the LCF of LCF–Cu _120–300_. The LCF was separated from the Cu foil by a practical method. Briefly, a poly (methyl methacrylate) (PMMA) thin film was first coated onto the surface of the LCF by spin coating, and then PMMA-supported LCF was obtained by etching off the Cu foil with an ammonium persulfate solution [28]. Finally, LCF sheets were obtained by removing the PMMA support from the composite film using acetone. The obtained LCF sheets were picked up using a Cu mesh for TEM characterization. An LCF sheet (a few tens of square micrometers in area), which looked like transparent silk, covering the Cu grid was observed in the low-magnification TEM image, as shown in Figure 2a. The HRTEM images of LCF–Cu _120–300_ are displayed in Figure 2b, a multilayer ordered structure with 0.34 nm lattice spacing was observed [28]. Furthermore, the SAED image (Figure 2c) of the LCF sheet indicated that the diffraction dots were fully indexed to the hexagonal graphite crystal structure [29], proving the highly graphitized crystalline structure of the LCF [30]. Therefore, it was claimed that the LCF is mainly composed of multi-layer graphene-like lignin-based carbon sheets with high thermal conductivity [3]. The thickness of the LCF transferred onto a SiO_2_ sheet was measured as ~80–100 nm by AFM (Figure 2d), indicating that the LCF–Cu foil composites with nanoscale LCF were successfully achieved in this work. Figure 2e shows a curtain of ripples was observed and its height was measured as ~20–35 nm which was consistent with SEM analysis.

To summarize, through the SEM, TEM and AFM analysis, we showed that the LCF structure was dense and complete, and TEM results showed that the graphitization degree of the LCF was high when the RET was 120 min and the CT was 300 min. As is well known, the increase of graphitization has a decisive effect on the increase of thermal conductivity. Therefore, XPS and Raman analysis were used to further study the graphitization degree of LCF with different RET and CT.

#### 3.1.3. XPS Analysis

**Figure 3:**
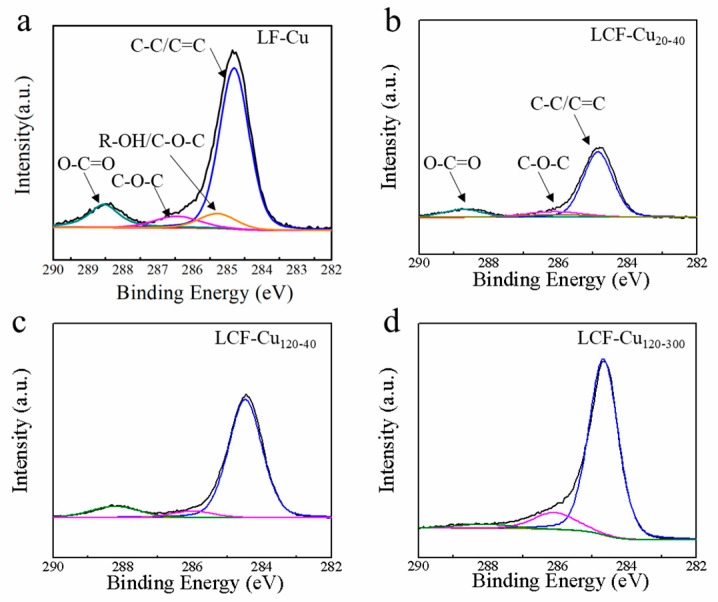
High-resolution XPS spectra of (**a**) LF-Cu, (**b**) LCF–Cu _20–40_, (**c**) LCF–Cu _120–40_, and (**d**) LCF–Cu _120–300_.

The changes in the surface chemistry of the samples were investigated by XPS through de-convoluting the peak of C 1 s, as shown in Figure 3a–d. The deconvoluted C 1 s spectrum of lignin is shown in Figure 3a. The peaks at 284.6, 285.3, 286.2, and 288.4 eV were respectively assigned to C–C/C=C, R–OH/C–O–C, C–O–C and O–C=C bonds [31]. All LCF–Cu samples present similar peaks at 284.6, 286.2, and 288.4 eV which were respectively assigned to C–C/C=C, C–O–C, and O–C=C bonds [31]. This result suggested that the extension of RET and CT during the annealing treatment hardly changed the bond types of carbon atoms in the LCF, except for a change in the relative contents of these bonds. The concentration of the surface carbon of the samples was calculated from the XPS spectra after correcting the relative peak areas with sensitivity factors (Figure 3b–d). Generally, annealing in an inert gas atmosphere causes deoxygenation and dehydrogenation of lignin [32]. As expected, after annealing at 1000 °C for 20 min (RET) and cooling over 40 min (CT), compared to lignin (59.4%), the relative carbon content of LCF–Cu _20–40_ was increased to 67.1% (Table 1). As the RET was increased to 120 min, a higher relative carbon content of 68.9% was observed in LCF–Cu _120–40_. Thereby, the highest relative carbon content of 88.0% was observed in LCF–Cu _120–300_, as the longest RET and CT of 120 and 300 min, respectively, were employed, which suggested that the graphitization degree of the LCF was increased as RET, especially CT, was elongated (Table 1).

#### 3.1.4. Raman Analysis

**Figure 4:**
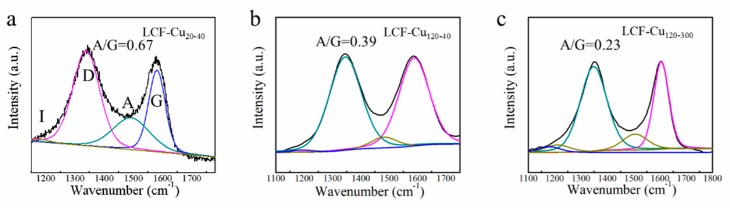
Raman spectra of (**a**) LCF–Cu _20–40_, (**b**) LCF–Cu _120–40_, and (**c**) LCF–Cu _120–300_.

Figure 4a–c show the Raman spectra of LCFs containing two remarkable bands at ~1350 and 1580 cm^−1^, which were assigned to D and G bands of carbon, respectively. The intensity of D band is related to the disordered structures of the sp^3^ carbon, while G band is related to the graphite lattice. The de-convoluted Raman spectra of the LCFs consisted of an impurity band (I) at 1140 cm^−1^ [33,34], a disordered graphitic band (D) at 1350 cm^−1^ [34,35], an amorphous band (A) at 1484 cm^−1^ [35,36], and an ordered graphitic band (G) at 1585 cm^−1^ [34,35]. The intensity ratio of A/G was used as an indicator of the graphitization degree, according to the previous study [33]. The A/G values of LCF–Cu _20–40_, LCF–Cu _120–40_, and LCF–Cu _120–300_ were respectively 0.67, 0.39, and 0.23, indicating the significant overall increases in the graphitization degree of LCF with the increasing RET and CT, which is corroborated by the increasing trend in bonding details of carbon atoms determined from XPS analysis. Furthermore, the appearance of the intensive D and G bands of LCF–Cu _120–300_ indicated the emergence of a two-dimensional (2D) crystalline lattice structure in LCF [36], which was also confirmed by the HRTEM images of LCF–Cu _120–300_ shown in Figure 2b. Presence of the 2D highly crystalline lattice in LCF guaranteed low restrictions for the phonon-assisted thermal transport. Meanwhile, previous studies have proven that using metals such as copper as the base can promote the graphitization degree of biomass during the carbonisation process [26]. Conclusively, the increasing RET and CT not only prolonged the graphitization process of lignin, resulting in deoxygenation and dehydrogenation, but also extended the catalytic graphitization of lignin under the catalysis of Cu foil [37], eventually improving the graphitization degree of LCF.

### 3.2. Effect of Annealing Conditions on Thermal Conductivity of Lignin-Based Carbon Film-Copper Foil Composite

#### 3.2.1. “Laser Flash” Technique Analysis

**Figure 5:**
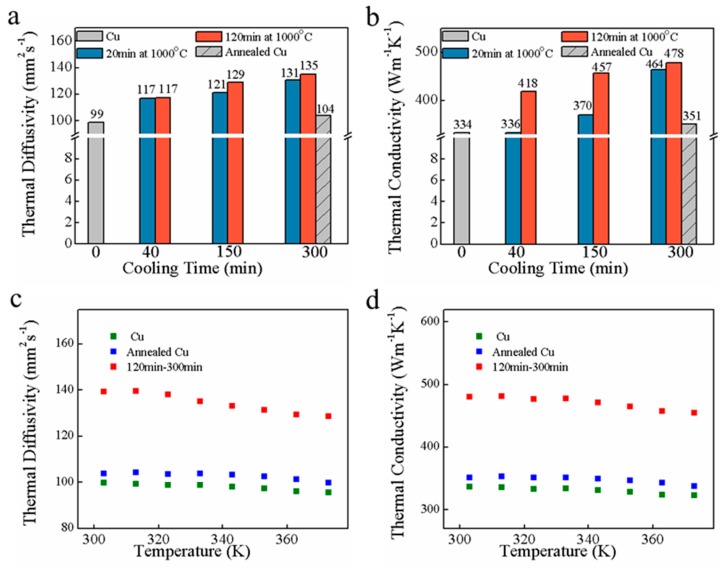
(**a**) α and (**b**) K of the untreated Cu foil, annealed Cu foil, and LCF–Cu sample at 333 K; (**c**) α and (**d**) K variations with the temperature of the untreated Cu foil, annealed Cu foil, and LCF–Cu _120–300_.

LCF was a dense and highly graphitized carbon film. This characteristic was beneficial to heat transfer of composites. Therefore, the K and infrared thermal imaging were used to study the capability of heat transfer of LCF–Cu. The K of the samples was investigated using the “laser flash” technique (LFT). The in-plane α of the sample was directly measured by the LFT. The K of the samples was then calculated by the following equation [2,22]: K = ραC_p_, where ρ (Table S2) is the mass density of the sample, C_p_ is the specific heat capacity of the sample measured by DSC (Table S1 and Figure S2), the C_p_ of untreated and annealed Cu foils are achieved from the reference database [38]. Figure 5a,b presents the in-plane α and K of untreated Cu foil, annealed Cu foil, and LCF–Cu film at 333 K. The α and K values of untreated Cu foil are 99 mm^2^ s^−1^ and 334 W m^−1^ K^−1^, respectively. During the high-temperature annealing, the grain size of Cu foils increased due to recrystallisation, leading to reduced defect density and improved thermal properties of Cu foils [4]. Thereby, the annealed Cu foil exhibited higher α and K of 104 mm^2^ s^−1^ and 351 W m^−1^ K^−1^, respectively, as compared with the counterparts of untreated Cu foil. By combining the excellent thermal property of the crystalline LCF, the compositing of LCF with Cu foil resulted in substantial increase in α and K of LCF–Cu _120–300_ as compared to those of a Cu foil annealed under the same conditions. Comparison of the properties of the LCF–Cu film samples indicates that α and K increase with the extension of the RET and CT, mainly due to the increased graphitization degree of LCF, which contributes to the phonon thermal transport resulting in the improvement of heat conduction performance [39]. LCF–Cu _120–300_, which was subjected to annealing treatment with the longest RET of 120 min and CT of 300 min, exhibited outstanding α and K values of 135 mm^2^ s^−1^ and 478 W m^−1^ K^−1^, respectively, which are 37% and 43% higher than those of the untreated Cu foil, respectively. It is worth noting that, although the α of LCF–Cu _20–40_ is higher than that of pure Cu foil both before and after annealing treatment, the high amorphous degree of LCF in LCF–Cu _20–40_ led to a slightly higher K than that of the untreated Cu foil, but lower than that of annealed pure Cu foil, which suggested the still restriction of phonon thermal transport by the relative high disorder of LCF in LCF–Cu _20–40_. These results indicated that the combination of highly graphitized LCF and the increased grain size of Cu foil under the annealing treatment with prolonged RET and CT dramatically promoted the heat conduction performance of the composite LCF–Cu film.

The variations in α and K with changing temperature of the untreated Cu foil, annealed Cu foil, and LCF–Cu _120–300_ are shown in Figure 5c,d. Generally, the α always declined with the increasing temperature [38]. Therefore, it was observed that α and K values of the sample declined with increasing temperature, which was explained by the intrinsic and boundary-scattering mechanisms of the heat carriers [40]. Goli et al. demonstrated that Cu foils capped with single-layer and multilayer graphene by CVD method increased their in-plane K by up to 24% near room temperature [4]. In this work, the in-plane K of LCF–Cu _120–300_ was found to be ~43% higher than that of the untreated pure Cu foil. More importantly, the K value of the prepared LCF–Cu composite film is comparable to those of the reported Cu-based thermal management materials (Table 2) such as pure Cu foil (313 W m^−1^ K^−1^) [4], graphene/Cu/graphene composite film (376 W m^−1^ K^−1^) [4], composite of graphene nano-platelet and Cu (525 W m^−1^ K^−1^) [2], carbon nanofiber-Cu composite (435 W m^−1^ K^−1^) [1], and Cu-graphite-Cu sandwich (527 W m^−1^ K^−1^) [41]. Consequently, the current investigation revealed that the K of copper foil was effectively improved by lignin-based carbon film which was prepared with lignin, the pulping mill waste, through an easy and practical method.

#### 3.2.2. Thermal Infrared Imaging Analysis

**Figure 6:**
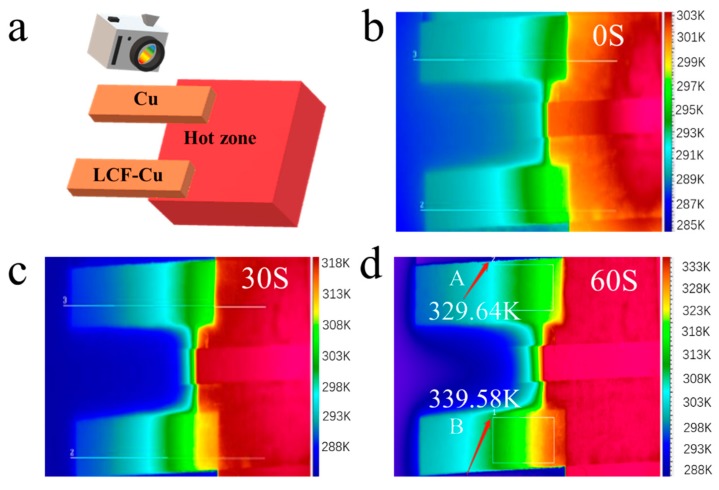
(**a**) Sample placement for infrared imaging. Continuous infrared imaging results: at 0s (**b**), 30s (**c**), and 60s (**d**).

The infrared images of the samples which were characterized by the thermal emission microscope are demonstrated in Figure 6. The schematic of sample placement for infrared imaging is shown in Figure 6a, where the top part is the untreated Cu foil and the bottom part is the LCF–Cu _120–300_. The untreated Cu foil and LCF–Cu _120–300_ were heated on the right side by the heater panel, and heat was then transferred to the left side which was exposed to the surrounding atmosphere (~298 K). The infrared images of the sample were captured at 30 s intervals with the temperature increase of the heater panel from 298 K to 343 K within 60 s (Figure 6b–d) to study the difference between the capability of heat transfer of the untreated Cu foil and LCF–Cu _120–300_. The results indicated that the surface temperature of the untreated Cu foil (position A in Figure 6d) was 10 K lower than that of the LCF–Cu_120–300_ (position B in Figure 6d) when the temperature of the heater panel was 343 K, indicating that superior heat conduction performance was achieved by the modification of the Cu foil with LCF. Therefore, the LCF–Cu composite film is highly promising as an efficient and low-cost heat conductive material in the thermal management application of modern electronics.

## 4. Conclusions

The LCF–Cu composite film with high K was fabricated by the easily achieved spin coating of lignin thin films on Cu foils followed by annealing treatment. With the increase of the RET and CT, the density of LCF tended to increase and graphitization was enhanced; at the same time the thermal conductivity of LCF–Cu has been improved. As a result, the K of all Cu foils modified by LCF was improved and the K of LCF–Cu _120–300_ was 43% higher than that of the untreated Cu foil. Infrared imaging also confirmed the outstanding heat-conducting performance of the LCF–Cu composite film. Therefore, the LCF–Cu composite film is highly promising as an efficient, safe, and low-cost thermal management material in advanced electronics. Furthermore, the carbon films prepared from lignin in this work has broadened the thinking of preparing advanced carbon materials from lignin.

## Figures and Tables

**Table 1:** Relative contents of elements and carbon species.

Sample	RT (min)	CT (min)	Relative Content (%) *					
			C	O	C–C/C=C	C–O–C	O–C=C	R–OH or C–O–C
Lignin	-	-	59.4	40.6	71.3	7.8	12.8	8.1
LCF–Cu_20–40_	20	40	67.1	32.9	74.8	12.8	12.4	-
LCF–Cu_120–40_	120	40	68.9	31.1	82.2	5.9	11.9	-
LCF–Cu_120–300_	120	300	88.0	12.0	84.3	12.0	3.7	-

* Relative % carbon species content = $\;\frac{A_{\mathrm{carbon}}}{A_{\mathrm{total}\;\mathrm{carbon}}}\times 100\%$, where A_carbon_ is the peak area of the specific carbon species, A_total carbon_ is the total peak area of carbon species.

**Table 2:** Comparison of various copper-based thermal management materials.

No.	Matrix	Method	Strengthening Phase	K (W m^−1^ K^−1^)	References
1	Cu	Carbonization	LCF	478	Current study
2	Cu	-	-	313	[4]
3	Cu	Chemical vapor deposition	Few layers graphene	376	[4]
4	Cu	Vacuum filtration and spark plasma sintering	Graphene nano-platelet	525	[2]
5	Cu	chemical mixing	Carbon nanofiber	435	[1]
6	Graphite	Electroplating Cu on synthetic graphite sheets	Cu	527	[41]

## Supplementary Materials

The following are available online at https://www.mdpi.com/2079-4991/9/12/1681/s1, Figure S1: SEM of the rapid evaporation of acetone led to a porous structure of the lignin film. Figure S2: The *Cp* values of LCF–Cu _120–300_ under different temperatures, Table S1: The *Cp* values of all samples, Table S2: The ρ of all samples, video: Video S1.
