# Supplementary material for: Fabrication of Lignin-Based Nano Carbon Film-Copper Foil Composite with Enhanced Thermal Conductivity
Source: Nanomaterials (Basel). 2019 Nov 25;9(12):1681. doi: 10.3390/nano9121681 (PMC6956031; doi:10.3390/nano9121681)
Supplement: Supplementary file 1 [file nanomaterials-09-01681-s001.zip › nanomaterials-647687-SI/SI.docx]

Supplementary Material

Fabrication of Lignin-Based Nano Carbon Film-Copper Foil Composite with Enhanced Thermal Conductivity

Bin Luo ^1,2^, Mingchao Chi ^1,2^, Qingtong Zhang ^1,2^, Mingfu Li ^1,2^, Changzhou Chen ^1,2^,
Xiluan Wang ^3^, Shuangfei Wang ^1,2^ and Douyong Min ^1,2,^*

^1^ College of Light Industry and Food Engineering, Guangxi University, Nanning 530004, China; luobinRobin123@163.com (B.L.); mingchaochi2018@163.com (M.C.); qingyutong110@163.com (Q.Z.); mingfuli@mail.gxu.cn (M.L.); chenchangzhou@gxu.edu.cn (C.C.); wangsf@gxu.edu.cn (S.W.)

^2^ Guangxi Key Laboratory of Clean Pulp & Papermaking and Pollution Control, Nanning 530004, China

^3^ Beijing Key Laboratory of Lignocellulosic Chemistry, Beijing Forestry University, Beijing 100083, China; wangxiluan@bjfu.edu.cn

***** Correspondence: mindouyong@gxu.edu.cn; Tel.: +86-0771-3949-947


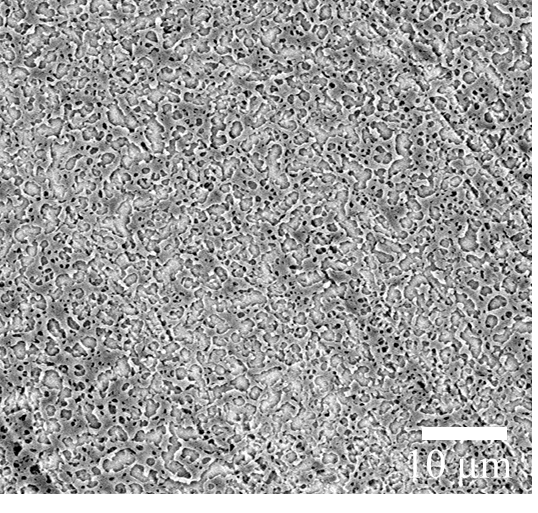


**Figure S1.** SEM of the rapid evaporation of acetone led to a porous structure of the lignin film.

**Table S1** The Cp values of all samples.

| Sample | Cp（J/gK） |
| --- | --- |
| Cu | 0.39 |
| Annealed Cu | 0.39 |
| LCF-Cu20-40 | 0.33 |
| LCF-Cu20-150 | 0.35 |
| LCF-Cu20-300 | 0.41 |
| LCF-Cu120-40 | 0.41 |
| LCF-Cu120-150 | 0.41 |
| LCF-Cu120-300 | 0.41 |





**Figure S2.** The Cp values of LCF-Cu 120-300 under different temperatures.

**Table S2.** The ρ of all samples.

| Sample | ρ(g/cm3) |
| --- | --- |
| Cu | 8.65 |
| Annealed Cu | 8.68 |
| LCF-Cu20-40 | 8.65 |
| LCF-Cu20-150 | 8.71 |
| LCF-Cu20-300 | 8.66 |
| LCF-Cu120-40 | 8.69 |
| LCF-Cu120-150 | 8.64 |
| LCF-Cu120-300 | 8.62 |
